# A rare case of primary ovarian pregnancy with diagnostic and surgical challenges: a case report

**DOI:** 10.1097/RC9.0000000000000397

**Published:** 2026-03-26

**Authors:** Aashik Jha, Abhishek Kumar Shah, Dev Kumar Shah, Bishal Khaniya, Dhirendra Yadav

**Affiliations:** a Maharajgunj Medical Campus, Institute of Medicine, Tribhuvan University Teaching Hospital, Kathmandu, Nepal; b Department of Obstetrics and Gynecology, Tribhuvan University Teaching Hospital, Kathmandu, Nepal

**Keywords:** adult female, beta human chorionic gonadotropin level, case report, ectopic pregnancy, lower abdominal pain, per vaginal bleeding, primary ovarian pregnancy

## Abstract

**Introduction and importance:**

Ovarian ectopic pregnancy (OEP) is a rare but serious condition, accounting for only 0.5–3% of all ectopic pregnancies. Because it is so uncommon and its symptoms often mimic other conditions, diagnosing OEP can be incredibly difficult.

**Presentation of case:**

This case report follows a 24-year-old woman, pregnant for the first time, who arrived at the hospital with abdominal pain and vaginal bleeding. Surprisingly, she had none of the typical risk factors associated with ectopic pregnancies. Initially, her condition was misdiagnosed until an ultrasound revealed a gestational sac in her adnexa. She underwent emergency laparotomy, which confirmed a ruptured OEP. Surgeons performed an ovarian wedge resection, and the diagnosis was later confirmed by histopathology.

**Clinical discussion:**

This case highlights how crucial early detection and surgical intervention are in OEP, especially in settings with limited medical resources. It also reinforces that any woman of reproductive age with sudden abdominal pain and bleeding should be evaluated for OEP, even if she has no known risk factors.

**Conclusion:**

Timely treatment and histopathological verification are essential to prevent complications and protect future fertility. This case also highlights the need for heightened awareness, rapid imaging, and decisive surgical action even when symptoms do not follow the usual pattern. Given that few documented cases exist, particularly in low-resource environments, this report adds valuable insights into the diagnosis and management of OEP.

## Introduction

Ectopic pregnancy is a complication of pregnancy in which an embryo attaches itself anywhere outside of the uterus. It is an obstetric emergency and a cause of pregnancy-related death in the first trimester of pregnancy^[^1^]^. Ectopic pregnancies occur in 2% of natural pregnancies, with 95% in the fallopian tubes and 5% in the ovary, cervix, or abdomen^[^2,3^]^. Ovarian ectopic pregnancy (OEP) is a rare but well-known pathology and constitutes approximately 0.5–3% of all ectopic pregnancy cases^[^4^]^.

It is a commonly occurring gynecological emergency and a leading cause of maternal mortality in the first trimester^[^1^]^. Due to lack of elasticity in the ovarian cortex, in most cases OEP gets ruptured. About 91% of OEP rupture during the first trimester, 5.3% end in the second trimester, and 3.7% end in the third trimester^[^2^]^. Only one case of an OEP that advanced to full-term delivery has been reported so far^[^5^]^.

HIGHLIGHTS

- A 24-year-old female presented with lower abdominal pain and per vaginal bleeding, later diagnosed with primary ovarian pregnancy.
- Per speculum examination of the cervix showed a healthy cervix, with no active bleeding and only some amount of vaginal secretions.
- Transabdominal ultrasound revealed 3.2 cm echogenic embryo-like mass in the right adnexa, and surgical exploration confirmed the diagnosis.
- The case highlights the need for a multidisciplinary approach to address even common symptoms with consideration of all possibilities.

The risk factors of OEP include intrauterine devices, assisted reproductive technology, and pelvic inflammatory disease (PID). The clinical manifestations of OEP, including amenorrhea, abdominal pain, and vaginal bleeding, have no obvious specificity compared with tubal pregnancy. Due to the high vascularity of ovarian tissue, OEP patients commonly develop intraperitoneal bleeding, which can progress to life-threatening hemorrhagic shock resulting from pregnancy sac rupture. The rupture rate in case of OEP is 86.61%, which is significantly higher than that of tubal ectopic pregnancy, which is 15.97%^[^5,6^]^.

As both OEP and tubal ectopic pregnancy generally present as adnexal masses, differential diagnosis between OEP and tubal ectopic pregnancy is challenging. Three-dimensional pelvic ultrasound and serum beta subunit of human chorionic gonadotropin (β-hCG) are widely used auxiliary tests. Detecting the location of unruptured OEP masses by ultrasound is possible; however, detecting the location of ruptured masses of OEP is difficult. A study reported that OEP patients had higher serum β-hCG levels than tubal pregnancy patients; however, due to rareness of the disease and limited data, this conclusion needs to be further validated by research with more cases^[^6,7^]^.

High levels of maternal morbidity and mortality exist due to the infrequency of OEP presentation and its diagnostic challenges^[^8^]^. Therefore, early recognition of clinical manifestations, timely laparoscopic confirmation of diagnosis, and simultaneous surgical treatment have become the key to the management.

This case report has been reported in line with the SCARE checklist^[^9^]^.

## Timeline

The patient developed abdominal pain on 10 May 2025 and visited our center on the 12th day, i.e., 22 May 2025. She underwent investigations by 24 May 2025 and finally was operated on 27 May 2025. She was discharged on 30 May 2025 and had her follow up on 2 June 2025.

## Patient information

### Demographic details

A 24-year-old woman who was a housewife by occupation.

### Presentation

She presented with 2 months of pregnancy, presented to the emergency department of our hospital with chief complaint of lower abdominal pain for the past 12 days, and per vaginal (PV) bleeding that lasted for 2 days, which occurred 10 days before admission.

The patient was in her usual state of health until 12 days prior to admission, when she developed a sudden onset of lower abdominal pain localized to the suprapubic region. The pain was pricking in nature, mild to moderate in intensity, radiated throughout the abdomen, and was relieved with rest and medications. She also experienced PV bleeding, which lasted for 2 days and required two pads per day. This was associated with two episodes of vomiting. Initially, she visited a local clinic, where she was diagnosed with cystitis and prescribed oral medications. However, her symptoms persisted, and she was admitted to local hospital for 3 days, where no significant improvement was seen. She was then referred to our hospital, where further investigations were conducted, and she was diagnosed with right ruptured ectopic pregnancy.

### Past medical and surgical history

The patient had no history of hypertension, diabetes mellitus, cardiac disease, or any other chronic illness. The patient is a non-smoker and non-alcoholic and follows a mixed diet. There were no previous history of any surgery and no history of miscarriages, abortion, or any gynecological illness. No such episodes were noted previously.

### Drug and allergic history

No any regular medications or allergies seen.

### Family history

No significant family history of any chronic illnesses, congenital abnormalities, or gynecological disorders.

### Menstrual history

The patient attained menarche at 14 years of age. She has a regular menstrual cycle of 4-5 days occurring every 28–30 days. Her last menstrual period (LMP) was 50 days before the visit. She had not been using any contraceptive methods before conception.

## Clinical findings

On physical examination, the patient was in fair condition, well oriented to time, place, and person. No signs of acute distress were noted. Her vitals were stable, with a pulse rate of 88 beats per minute, blood pressure of 110/70 mmHg, and temperature of 98.6°F. She was afebrile, without pallor, lymphadenopathy, or edema. On systemic examination, findings were unremarkable, with bilateral equal air entry in the lungs and normal heart sounds (S1 and S2) without murmurs.

On abdominal examination, inspection was unremarkable. On palpation, the lower abdomen was rigid, with voluntary guarding. Marked tenderness and rebound tenderness were elicited in the right iliac fossa. There were no palpable masses. On per speculum (P/S) examination, the cervix appeared healthy with no active bleeding, but vaginal discharge was present. On per vaginum examination, the uterus was bulky and retroverted. A 3 × 3 cm mass was palpated in the right fornix, and cervical motion tenderness (CMT) was present.

## Diagnostic assessment

On investigations, hemoglobin was found to be 11.3 mg/dL with all routine hematological and biochemical parameters within normal range. The blood β-hCG level was 1700 mIU/mL (normal <5 mIU/mL), which is a significant increase.

**Figure 1.**
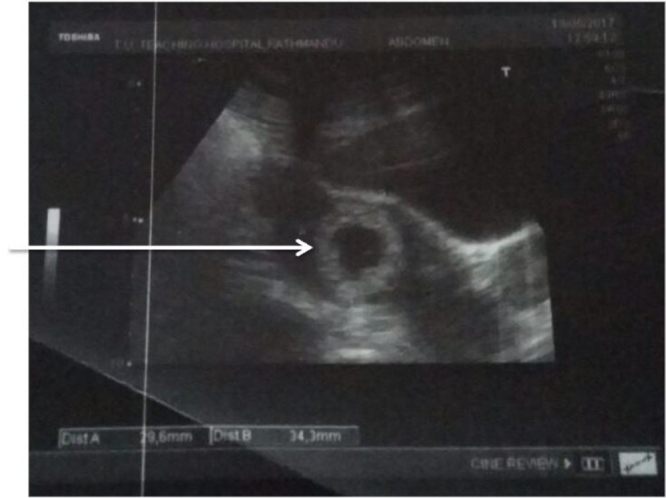
USG finding showing normal uterus but echogenic structure in right adnexa.

A transabdominal ultrasound (USG) of the abdomen and pelvis was performed as an initial assessment according to our hospital protocol. It showed uterus of normal size (8.3 × 3.8 × 5 cm) (Fig. 1), with an empty endometrial cavity. A gestational sac measuring 3.2 cm was noted, containing an echogenic embryo-like structure and a yolk sac in right adnexa. No fetal cardiac activity was observed. Minimal free fluid was present adjacent to the gestational sac. The USG findings were suggestive of right ectopic pregnancy. Transvaginal USG was not done since the transabdominal USG already showed the ovarian pregnancy and it needed urgent intervention.

An emergency exploratory laparotomy was planned suspecting rupture of ovarian pregnancy. It was performed under general anesthesia at 7 weeks of gestation.

**Figure 2.**
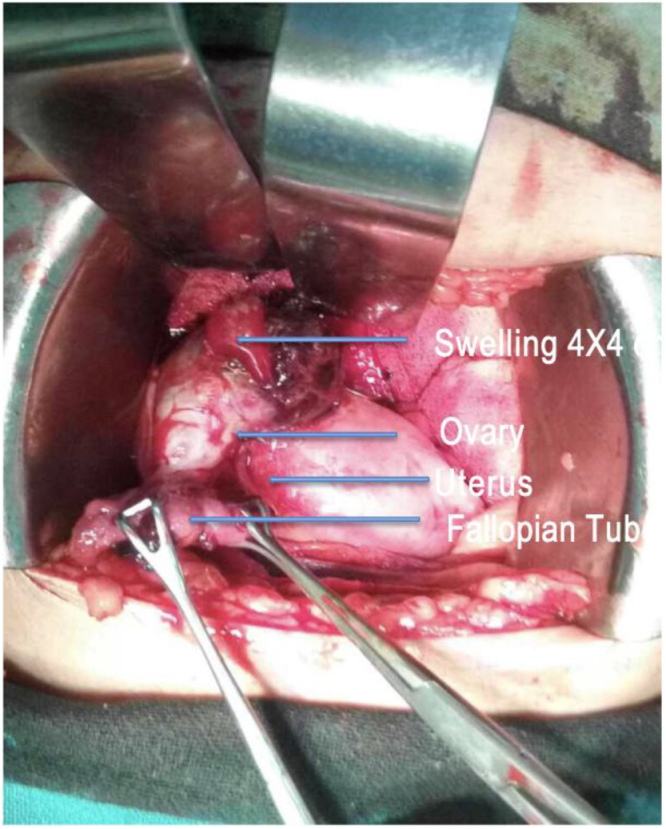
Intraoperative picture showing a swelling distal to edge of right ovary with the swelling of 4 × 4 cm.

**Figure 3.**
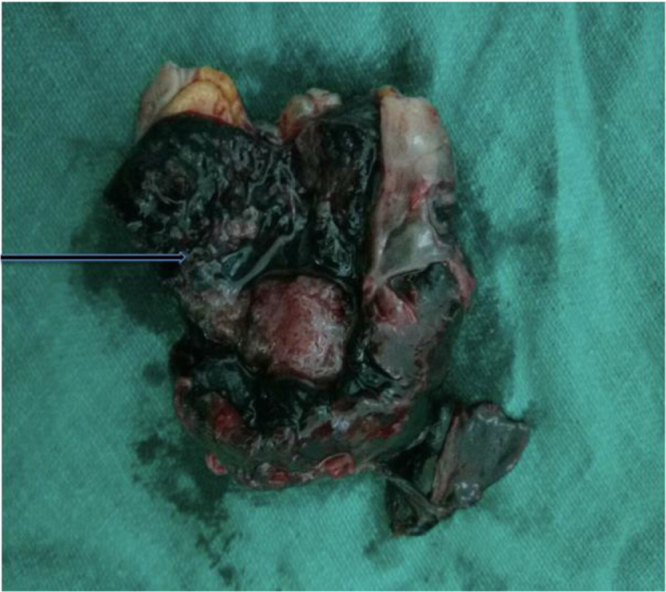
Dissection of the mass inside the swelling.

Intraoperatively, approximately 100 mL of blood and 50 g of clots were found in the peritoneal cavity suggestive of hemoperitoneum. A 4 × 4 cm swelling was noted on the distal edge of the right ovary, covered by clots with active bleeding from one point. Right fallopian tube was swollen, extending into the mesovarium (Fig. 2). Wedge resection of the right ovary was performed with approximately 30% of ovarian tissue preservation. The wedge section on examination revealed a product of conception-like material along with blood clots, highly suggestive of a ruptured right OEP (Fig. 3).

Following surgery, the hemoglobin level was found to be 10.9 gm/dL, all routine biochemical and hematological parameters were within normal limits, and vitals were stable along with decreasing level of β-hCG.

**Figure 4.**
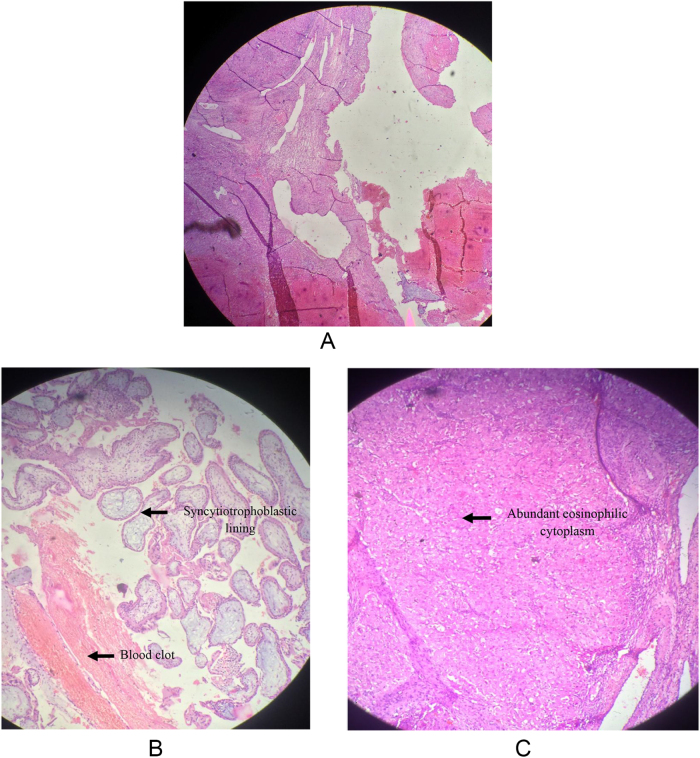
(A) Histopathologic slide [hematoxylin and eosin (H&E) staining 200×] showing trophoblastic tissue with chorionic villi and ovarian stroma. (B) Histopathology slide (H&E staining 200×) showing syncytiotrophoblastic lining and blood clot in the ovary. (C) Histopathology slide (H&E staining 200×) showing trophoblastic tissue with abundant cytoplasm.

The histopathological examination of the excised ovarian tissue revealed the presence of chorionic villi lined by trophoblastic cells along with blood clots. There was no evidence of hydropic changes in the chorionic villi. Adjacent ovarian tissue showed corpus luteum (Fig. 4a–c). The histopathological evaluation confirmed the diagnosis.

### Follow-up and outcomes

The hospital stay was uneventful with no significant complications. She was discharged on the 4th postoperative day with advice to follow up on the 7th day for suture removal.

## Reasons for discussion

OEP is one of the rarest forms of ectopic pregnancy, accounting for almost 3% of all the ectopic pregnancies^[^10,11^]^. Its incidence ranges from about 1 in 7000 to 1 in 40 000 pregnancies^[^12^]^. It is often misdiagnosed as ruptured tubal ectopic pregnancy or ruptured hemorrhagic luteal cyst due to similar presentation and USG findings. In a study of 25 ovarian pregnancies, Hallatt found that only 28% of cases had a correct surgical diagnosis. In the remaining cases, pathologists made the diagnosis^[^12,13^]^.

Prior to surgery, the diagnosis is challenging because the clinical manifestations are similar to those of tubal pregnancy, which can include abdominal pain, irregular vaginal bleeding, amenorrhea, and an adnexal mass^[^14^]^.

According to a study by Razeiel *et al*, 90% of ovarian pregnancies among contraceptive intrauterine device (IUD) users occurred in the past, so a history of recent IUD use combined with an extrauterine pregnancy should be regarded with suspicion regarding the likelihood of an ovarian pregnancy^[^15^]^.

## Discussion

Depending on the location of the fertilized ovum in the ovary, ovarian pregnancy can be classified as primary or secondary OEP. Primary ovarian pregnancy occurs when an ovum that has not yet been released from the ovary is fertilized, or when a fertilized ovum undergoes primary implantation in the ovary following reverse migration from the fallopian tube. Secondary ovarian pregnancy occurs when the gestational sac is re-implanted on the ovary surface following a tubal abortion or rupture. Primary OEP can be further intrafollicular or extrafollicular. In the extrafollicular, fertilization takes place when the fallopian tube’s egg is released and replaced in the ovarian stroma, whereas the intrafollicular type refers to the failure of follicular ovulation in which the fertilized ovum is deposited in the early corpus luteum and develops in the ovary where the ovarian tissues completely encapsulate the embryo^[^16^]^. The incidence of OEP ranges from 1 in 7000 to 1 in 40 000 live births^[^10^]^, making it a very rare phenomenon. In summary, the overall risk of an ovarian pregnancy varies from 0.014% to 0.0025% for pregnant women and from 0.5% to 1% for women who have symptoms or an adnexal mass that raise the possibility of an ectopic pregnancy^[^17^]^.

### Proposed hypothesis for OEP

The exact mechanism is unknown. The intrafollicular pregnancy is hormonal, resulting in a trapped ovum inside the follicle. The thickened ovarian tunica albuginea is another hypothesized cause. It can also be the consequence of ineffective ovum pick-up caused by the inefficient sweeping of fimbria ovarica across the ovary surface. Delay in ovum release and tubal malfunction can also be responsible.

#### Risk factors

The major risk factors of OEP include history of IUD use, PID, sexually transmitted infections, use of assisted reproductive technologies, prior pelvic surgery, endometriosis, previous ectopic pregnancy, salpingitis, advanced maternal age, multiparity, and, more rarely, infertility^[^4,18,19^]^.

An IUCD is the most significant risk factor, accounting for up to 57–90% of patients with primary OEP. The theory behind this is that although the IUD provides protection from intrauterine implantation, it does not prevent ovarian implantation. Specifically, it is thought that the IUD may potentiate ovarian implantation due to changes in prostaglandin synthesis that subsequently increases tubal peristalsis^[^2,20,21^]^. Berger and Blechner documented the ratio of OEP among women using IUD to be 1:9 and prevalence in general population to be 1:150–200^[^22^]^. Our case had no history of IUD use.

Cases of ectopic ovarian pregnancy have also been reported, which occurred after subtotal hysterectomy^[^23^]^. More rarely, ovarian pregnancy can occur without the presence of the classic risk factors as described in case of Flystra^[^23^]^. No other risk factors were linked to our patient. In contrast to tubal ectopic pregnancy, OEP is not linked to subfertility or PID.

### Clinical presentations

Abdominal pain and vaginal bleeding, or menstrual disorders are the most common clinical symptoms of ovarian EP. If these symptoms occur along with a history of prior amenorrhea, a high β-hCG level, and an ultrasound showing no gestational sac in the uterine cavity, an ovarian EP should be evaluated^[^24^]^. The most significant USG findings in OEP is the gestational sac or cystic structure, which is positioned on the ovary, is of different echogenicity, and in certain situations, even portions of the fetus are visible^[^25^]^. One of the most important challenges in OEP diagnosis is to differentiate it from ruptured corpus luteum, hemorrhagic ovarian cyst, or tubal pregnancy both during ultrasound or surgery. Therefore, diagnosing ovarian pregnancy based on clinical presentation alone is limited. Our patient presented with the typical symptom of abdominal pain along with PV bleeding.

### Diagnosis

The preoperative diagnosis of an ovarian pregnancy is not simple. The clinical symptomatology is not very different from that of a tubal pregnancy. The diagnosis is often made at surgery and requires histological confirmation. The ruptured early OEP can be mistakenly diagnosed as corpus luteum hemorrhage in many cases^[^13^]^. Macroscopic evaluation alone is insufficient and only indirect signs may be indicative. Definitive diagnosis must await histological examination in order to distinguish an intrafollicular pregnancy from a ruptured corpus luteum.

In 1878, Spiegelberg^[^26^]^ proposed criteria for the surgicopathological diagnosis of primary ovarian pregnancy. He stated that the fallopian tube on affected side must be intact, fetal sac should occupy the position of ovary, ovary must be connected to uterus by ovarian ligament, and the ovarian tissue must be located in sac wall. Although Spiegelberg’s criteria are still referred to in recent literature, they are of limited value for modern clinical practice. Recent studies suggest that the actual incidence could be up to 1 in 1400 deliveries if the criteria other than those of Speigelberg’s are taken into consideration^[^27^]^. However, in our case, the 4th criterion was modified as chorionic villi were detected without concurrent detection of an intact ovarian parenchyma.

Transvaginal ultrasonography in combination with quantification of β-HCG levels has significantly increased the accuracy of diagnosis. Transvaginal ultrasonography plays a key role in the preoperative diagnosis of an unruptured ovarian pregnancy, whereas in ruptured cases there are no typical ultrasonographic findings that could differentiate it from a ruptured tubal pregnancy or a ruptured corpus luteum cyst^[^28^]^.

According to Almahloul *et al*, most ectopic pregnancies were diagnosed within 8 weeks of amenorrhea, but 15% of pregnancies had a duration of up to 44 weeks. However, in 10 cases symptoms started before the end of a menstrual cycle (before 28 days of amenorrhea)^[^17^]^. Our patient was diagnosed after 7 weeks of amenorrhea.

### Preferred therapeutic procedure

Treatment of ovarian pregnancy is divided into two groups: surgical and conservative, depending on the time of diagnosis.

Methotrexate alone, or combined with Mifepristone, are the most commonly used medicine with the best therapeutic results. It can be used in early stage patients with hemodynamic stability^[^20^]^. Green-top criteria have been developed suggesting that the use of methotrexate can be considered if the following criteria are met: no signs of hemodynamic compromise, no evidence of blood in the pelvis, a pregnancy size of <3.5 cm with no fetal heart activity, and a β-hCG level of <3500 IU/L^[^29^]^. If used, a single dose of methotrexate by laparoscopic-assisted injection into the ectopic site is recommended. Higher failure rates have been reported if the gestational sac is >3.5 mm or if the serum β-hCG level is <5000 IU/L^[^30^]^. There are few recommendations for medical management to be appropriate when the risks of surgery are high. Medical treatment prevents ovarian tissue loss and pelvic adhesion and preserves fertility. Little evidence is available regarding medical management of OEP using methotrexate; however, some case reports have described successful treatment^[^20^]^. The American Society for Reproductive Medicine does not recommend methotrexate as the first-line treatment of OEP^[^30^]^.

Surgery is the mainstay of treatment for OEP. Laparoscopy is considered the gold standard for a definitive diagnosis and can be used to determine the location of bleeding and pregnancy as well as to direct the proper surgical treatment^[^31^]^. Depending on the size of the lesion, ovarian pregnancy lesion removal, ovarian wedge resection, or partial oophorectomy may be performed. Ovarian tissue is preserved as much as possible^[^32^]^. There was no significant difference found in reproductive outcomes between patients who underwent laparotomy and those who underwent laparoscopic surgery^[^6^]^. Laparoscopic surgery is the better option for OEP patients due to its benefits, which include a quick recovery after surgery and a brief hospital stay.

We preferred surgery over medical management in our case because the patient was symptomatic, with suspected ruptured ovarian pregnancy. During our laparotomy, an ovarian wedge resection was performed along with suturing and preservation of the remaining ovarian tissue. According to Hallatt’s analysis of 25 ovarian pregnancy cases, only 28% of them had the proper surgical diagnosis. In the remaining cases, the diagnosis was made by histopathology examination^[^13^]^. However, in our case, the suspected diagnosis was confirmed by histopathological examination. Adnexectomy or oophorectomy may be required in some cases with severe bleeding and a delayed diagnosis^[^13,33^]^. The recurrence rate of ovarian pregnancy is very low, the pregnancy rate after treatment is high, and there is no impact on female fertility^[^34^]^.

### Complications

Massive and potentially fatal bleeding may result from the ectopically implanted trophoblast’s close proximity to the ovarian and uterine vessels as well as the increased vascularity that occurs during pregnancy^[^4^]^. One study found that 80% of OEP had hemoperitoneum on ultrasound^[^35^]^ and another found that up to 30% presented with circulatory collapse^[^35^]^. Mortality rates are lower in areas where diagnostic and emergency surgical services are available, even though there is a higher volume of blood loss and a greater need for blood transfusions^[^30^]^. Occasionally, ovarian pregnancy may lead to choriocarcinoma^[^36^]^. Despite early diagnosis and treatment, which has significantly reduced morbidity and mortality, overall ectopic pregnancies are still responsible for 10% of deaths in the first trimester^[^37^]^.

## Conclusion

Despite being uncommon and dangerous, ovarian pregnancy must be understood in order to lower the morbidity and mortality rates that are linked to it. OEP should be considered as one of the important differential diagnoses in a female of reproductive age presenting with an acute abdomen, amenorrhea, and PV bleeding since ovarian pregnancies can occur before a delay in menstruation.

Diagnosing an OEP is difficult and is usually made intraoperatively, with confirmation by histopathology. It can occur without presence of any classical risk factors for ectopic pregnancy.

Although surgery is the preferred treatment for ovarian pregnancy, histological confirmation is still necessary for the diagnosis to be made. While ultrasound can identify ovarian gestations in cases where the tubal pregnancy has not ruptured, it is difficult to distinguish between an ovarian pregnancy and another tubal pregnancy in a ruptured state.

Medical management is not suggested due to lack of evidence. Laparoscopic surgery is a better choice than laparotomy for OEP treatment. OEP patients have a good reproductive prognosis. In addition to protecting the mother’s health, ensuring early detection and appropriate treatment is essential to minimizing complications that may compromise future fertility. Even in the absence of conventional risk factors, medical professionals should be acutely aware of the possibility of OEP, highlighting the unpredictable and individual nature of each patient’s clinical journey.

These four features made this an unusual case: the rarity of the case, lack of predisposing factors, diagnostic dilemma, and management modality.

## Strength and limitations

Here in the case we have described the step-by-step approach with relevant clinical findings.

We had a real challenge to actually diagnose the mass non-invasively, and our histopathological examination after history gave the confirmatory diagnosis.

## Data Availability

The data that support the findings of this study are available from the corresponding author upon reasonable request.
